# Alloys

**Published:** 1855-04

**Authors:** George Watt


### ALLOYS.

BY GEORGE WATT, M.D., D.D.S.,

Read before the Mississippi Valley Association, Feb. 23d, 1855.

Having been appointed to read an essay at this meeting of the association, I propose to offer you a few remarks on the general properties of alloys.

The term alloy, though once more limited, means a compound of any two or more metal. It is still a matter of dispute with some, whether metals combine chemically, or whether their particles are mechanically mixed, in the formation of alloys. It is known to all that some unite much more readily than others. Gold and platinum, for example, unite far below the melting point of the latter, while silver and iron are united with difficulty.

This subject, though somewhat neglected, is of great importance, as each alloy is, in reference to the arts and manufactures, a new metal, both on account of its physical and chemical properties. An extensive field here lies open. Only a few alloys have been studied by the chemist, and still fewer have been practically employed. Slight changes often constitute valuable improvements in these bodies, and there is no end to their modifications.

Metals do not unite with each other in the solid state. Assuming that alloys are chemical combinations this is easily explained, their affinity being counteracted by the force of cohesion, and if they are mechanical mixtures the case is equally clear, for the ultimate particles of the metals must be separated to some extent in order to commingle. Two metals will, in some cases combine, when but one of them is liquified, as when brass is formed by the immersion of copper in melted zinc.

Though metals appear to unite with each other in all proportions, yet it is probable that they tend to combine in a definite ratio, for several atomic compounds of this kind occur native. The native gold of the auriferous sands, for example, is an alloy with silver, in the ratio of one equivalent of silver, united with four, five, six and twelve equivalents of gold, but never with a fractional part of an equivalent. But the alloys being generally, as it were, soluble in each other, this definiteness of combination is not observable in most cases.

The alloys, like the metals, possess metallic lustre, conduct heat and electricity, are, to some extent, malleable, sonorous, elastic, and ductile, and are sometimes susceptible of crystallization. They often differ materially from the elements composing them. The hardness of a metal is generally increased by being alloyed; it is therefore frequently more elastic and sonorous. Alloys are generally less malleable and ductile than the metals composing them. Formed of two brittle metals, they are always brittle; of a ductile and brittle one, they are generally brittle, and are sometimes so when formed of two ductile metals.

The density of an alloy may be either greater or less than the mean density of its elements. Alloys of gold, with tin, zinc, bismuth, antimony or cobalt are examples of the former; those of gold with silver, copper, lead or iron, of the latter.

The fusibility of metals is greatly increased by alloying, as is well illustrated by the alloy of platinum and arsenic, and the well known alloy composed of 8 parts of bismuth, 5 of lead and 3 of tin, which melts at 212° instead of 514°, the mean of its elements. This increased fusibility is of great practical importance in the arts, and of special use to the mechanical dentist, as will be noticed presently.

The color of an alloy can not be correctly inferred from the color of its component metals. The alloys of copper with zinc and arsenic are familiar illustrations of this fact.

The tendency of metals to unite with oxygen is augmented by being alloyed, and this tendency is often, if not always, the result of galvanic action. According to Faraday, 100 parts of steel, alloyed with 1 of platinum, is dissolved with effervescence, in dilute sulphuric acid too weak to act with perceptible energy on common steel. This can only be explained by assuming that the steel is rendered positive by the presence of the platinum. The same thing is illustrated by the action of dilute acid on commercial zinc.

This tendency of the alloys to unite with oxygen, and the readiness with which metals oxydize, at their melting point, render some precautions necessary in the process of alloying. In combining tin and lead it is common to cover the surface of the metals with rosin, oil or other substance, having an affinity for oxygen greater than that of the metals. The same general principle, modified to suit circumstances, will answer in all cases.

There is also some difficulty in obtaining uniform alloys from metals differing much in specific gravity. Every dentist has observed this in gold alloyed with silver and copper. Adequate stirring, or re-melting is the remedy in such cases.

In uniting several metals, difficulty is often experienced from a want of affinity between two or more of them. This is best overcome, by uniting in pairs, those which combine most readily, and afterward uniting these with each other. For example, a small quantity of lead added to brass, produces a useful alloy for some purposes. This, however, would be difficult to accomplish directly, but it is readily effected by first combining the lead and zinc, and then adding melted copper.

Many alloys are important and interesting to the dentist. We will notice a few of these. Pure gold is too soft and flexible for the purposes of mechanical dentistry. This condition is overcome by alloying it with platinum, silver, copper, &c. In alloying for plate, some prefer silver, some copper, and others, as Prof. Harris, a mixture of both. A very good plate is obtained by alloying pure gold, (or ordinary coin,) with equal parts of silver and copper, reducing the gold to the required carat. Gold alloyed with platinum is strong and elastic, and is therefore valuable for clasps and backings, a less amount of substance affording the requisite strength. One part of platinum to 10 or 12 of gold will, in general, give satisfactory results.

The increased fusibility of metals when alloyed, enables us to unite two metals, or different pieces of the same metal, by a process called soldering. In the preparation of solders, several points require attention. They must be more fusible than the metal, or metals, to be united, and must be composed of metals having a strong affinity for those to be soldered together. Each metal, therefore, to a certain extent, requires a solder peculiar to itself. A very convenient though not a very fine gold solder is made by adding to any quantity of American coin, one-third its weight of silver and one-fourth its weight of copper. This is about the same as Harris's No. 1, and answers all ordinary purposes. Many formulas might be given, but those wanting them know where to get them.

Silver solder is the only alloy of that metal of use to the dentist. If silver plate be used, as it may be for temporary work in healthy mouths, the metal should be pure. This may be soldered with silver coin, or with any of the finer silver solders.

Some of the alloys of copper are interesting and useful to the mechanical dentist. Of these we may mention the ancient bronze, composed of tin, and copper; and brass, the well known alloy of copper with zinc. The former of these makes an excellent male cast, but the high temperature at which it fuses, and its affinity for oxygen, at the melting point, render it rather inconvenient for ordinary use. From its hardness, the plate will be "driven home" without any flattening of the prominences on the ridge. When used, it should be melted under rosin, powdered charcoal, pine sawdust, or some similar combustible substance, and, like all other alloys, should be taken from the fire and poured as soon as fused.

Brass, when pure, consists of 63 parts copper to 32 of zinc, being a definite compound, composed of 1 equivalent of zinc and 2 of copper. The brass of commerce is an uncertain compound, containing tin, lead and other alloys. This is a convenient substance in the laboratory for various incidental purposes not directly pertaining to mechanical dentistry.

Cheap and serviceable blow-pipes are constructed from it, and every dentist should be skilled enough to make his own, as a majority of those in market are unfit for use. It may be soldered with silver coin, any of the silver solders, or the common brass solder, composed of 2 parts brass, 1 of zinc, and a minute quantity of tin. It is sometimes used for casts, but is not reliable, on account of its shrinkage in congealing and cooling.

Commercial zinc is an alloy of zinc with iron, lead, cadmium, arsenic, &c. On account of its cheapness, and because it shrinks but little in congealing and cooling, it is much used for casts. It answers a tolerable purpose for ordinary cases, but its shrinkage is often a serious annoyance. The thing needed for casts is a convenient and cheap metallic substance which *expands*, in congealing, precisely as much as it shrinks in cooling from the point of congelation to the ordinary working temperature. An alloy composed of 4 parts of zinc to 1 of tin, fulfills these indications. It forms a very hard cast, and may be immersed in lead without the ordinary protection of whiting or lamp-black. Of the alloy of zinc with copper we have already spoken.

Equal parts of tin and lead make a good alloy for female casts. It is harder than either of its constituent metals, melts at a lower temperature than either of them, and as it shrinks less in cooling, gives a more correct counter cast. The attention of the profession was directed, some years ago, by Dr. Van Emon, to an alloy of tin and antimony, composed of, perhaps, 5 or 6 parts of the former to 1 of the latter. This is a good article for casts, can be used with lead for the female casts, and shrinks but little, if any, in cooling. It is easily oxydized, and should therefore be fused with the proper precautions, and taken from the fire as soon as melted. From our own experience, we consider the zinc and tin alloy above rerfered to, preferable to this or to any other we have used.

Two parts of lead, one of copper, and one of antimony, is the formula for Lassaigne's type metal. It makes a tolerable cast, expands slightly in congealing, but is rather brittle.

Of the alloy of platinum with gold we have already spoken, With steel it forms a tough strong alloy, an excellent material for excavators and pluggers. The best proportion is said to be 1 part of platinum to 100 of steel. None of its other alloys are of importance to the dentist.

The only remaining alloys interesting to the dentist, that we now think of, are the mercurials. Of these we have nothing to say at present, and we hope nothing farther need be said. Our views on the subject are known to the Society. We hope the whole matter may rest.

